# A New Reliability Coefficient Using Betting Commitment Evidence Distance in Dempster–Shafer Evidence Theory for Uncertain Information Fusion

**DOI:** 10.3390/e25030462

**Published:** 2023-03-06

**Authors:** Yongchuan Tang, Shuaihong Wu, Ying Zhou, Yubo Huang, Deyun Zhou

**Affiliations:** 1 School of Microelectronics, Northwestern Polytechnical University, Xi’an 710072, China; 2 School of Computer Science, Fudan University, Shanghai 200438, China; 3 School of Electronics and Information, Northwestern Polytechnical University, Xi’an 710072, China; 4 School of Engineering, University of Warwick, Coventry CV4 7AL, UK

**Keywords:** Dempster–Shafer evidence theory, conflict data fusion, evidence reliability coefficient, uncertain information fusion, uncertainty measure

## Abstract

Dempster–Shafer evidence theory is widely used to deal with uncertain information by evidence modeling and evidence reasoning. However, if there is a high contradiction between different pieces of evidence, the Dempster combination rule may give a fusion result that violates the intuitive result. Many methods have been proposed to solve conflict evidence fusion, and it is still an open issue. This paper proposes a new reliability coefficient using betting commitment evidence distance in Dempster–Shafer evidence theory for conflict and uncertain information fusion. The single belief function for belief assignment in the initial frame of discernment is defined. After evidence preprocessing with the proposed reliability coefficient and single belief function, the evidence fusion result can be calculated with the Dempster combination rule. To evaluate the effectiveness of the proposed uncertainty measure, a new method of uncertain information fusion based on the new evidence reliability coefficient is proposed. The experimental results on UCI machine learning data sets show the availability and effectiveness of the new reliability coefficient for uncertain information processing.

## 1. Introduction

Uncertainty management has been applied widely in practical engineering, such as sensor data fusion, pattern recognition and so on [1,2,3,4]. The new technology helps the information obtained in and out of the system to be integrated to make correct judgments. Bayesian probability theory, Dempster–Shafer evidence theory (D-S evidence theory), fuzzy structure and some other methods [5,6,7] have been used to enhance the ability to process uncertain data [8,9,10]. Among them, the Dempster–Shafer theory has an outstanding performance in uncertain information modeling and fusion in practical applications, such as multi-sensor target recognition [11], industrial alarm system design [12], web news extraction [13] classification with uncertainty [14,15,16], clustering [17,18,19] and so on [20,21]. The research on the Brain–Computer Interface also expanded the application of D-S evidence theory in the medical field [22].

The advantage of the D-S evidence theory lies in its characteristics of non-learning and no need for prior information [15,23]. Due to different sources of evidence, in the application process, the obtained evidence may have a high degree of conflict, resulting in counter-intuitive results after data fusion [24,25]. Many methods have been put forward to improve the performance of information fusion after Zadeh pointed out the shortcomings in the Dempster combination rule [26]. In order to analyze the rationality of the data fusion method, Liu characterized the feasibility of the classical Dempster rule with the two-dimensional form of conflict coefficient and distance between betting commitments between two BPAs [27]. Deng et al. proposed the generalized basic probability assignment (GBPA) generation method in the generalized evidence theory with a new combination rule [28]. If the belief value of the empty set m(∅) equals 0, GBPA degenerates into classic basic probability assignment (BPA). An et al. introduced a fuzzy reasoning mechanism into a similarity measurement model [29]. Many works proposed different methods to modify the combination rule [30,31,32]. In the classical method from Yager [33], it assumes that the frame of discernment (FOD) is closed and allocates the conflicting part of the belief value to the universal set of FOD, denoted as m(Θ).

A promising perspective of addressing conflict data fusion is using an uncertainty measure to manage the conflict in evidence [34,35,36]. Deng studied information entropy in the evidence theory and proposed Deng entropy, which has been applied widely [37]. Xiao proposed an information fusion method based on belief entropy and applied it to sensor data fusion in an uncertain environment [38]. A series of improved failure mode and effects analysis methods based on Deng entropy was proposed in [39,40]. The generation of a mass function with high reliability plays a key role in uncertain information processing with the D-S evidence theory [41]. There are also many methods of preprocessing different sources of evidence in D-S evidence theory, such as different concepts of evidence reliability [42,43], consistent strength [44], evidence reasoning [45,46] and uncertainty measure of negation evidence [47]. It seems that these preprocessing methods continue the idea of calculating the average arithmetic value of conflict evidence with different strategies [48].

There are many discussions on the use of the belief function in D-S evidence theory, such as the concept and redefinition of the conditional belief function [49,50] and the applicability of the belief function in specific situations [51,52]. The belief function is the result of assigning a probability to a set of evidence, not for a single proposition, and it is the probability assertion of the evidence based on knowledge [53]. If there is no evidence or initialization is required, the belief function should be assigned to all subsets of the FOD according to certain rules. In this case, the base belief function can be a solution [54]. The question is whether these pre-allocated belief functions have beneficial effects on evidence fusion all the time and how to avoid counter-intuitive combination results in some cases.

Most of the existing methods of improving D-S evidence theory have some limitations. First of all, the complexity brought by exponential-level evidence on the power set space is not considered enough. If the FOD is very large, not only is the calculation difficulty greatly increased but also the correlation among elements in the FOD is decreased or even lost. The conflict coefficient considers the factor product with an empty intersection between different sources of evidence, so the interaction among elements in the FOD should also be considered in the combination process. Secondly, there is a lack of substantive analysis of the original evidence. If the data are not processed according to the characteristics of the data itself, even if a more reasonable result is obtained in some cases, there may be a large deviation. To address the aforementioned open issues, this paper proposes a new evidence reliability coefficient.

The motivation of this study is to develop a new conflict data management method that can keep the original mathematical characteristics of the Dempster combination rule without adding more computation burden compared to most of the aforementioned methods of evidence preprocessing. The new evidence reliability coefficient defines the reliability coefficient through the differences in evidence sources from the prospect of using the Dempster combination rule. Inconsistencies between modified BPAs are eliminated. All elements in the FOD are assigned a belief value due to their inner connection.

The rest of this article is arranged as follows. Section 2 briefly introduces the Dempster–Shafer evidence theory and some existing conflict management methods. In Section 3, a new evidence reliability coefficient is proposed. Section 4 presents two experiments of applying the proposed method. Section 5 shows some discussions and open issues in this work. Section 6 is the conclusion of the work.

## 2. Preliminaries

### 2.1. Dempster–Shafer Evidence Theory

Assuming that the finite mutual exclusion set is Ω, the number of single-element subsets in the set Ω. Ω is referred to as the FOD and is defined as

(1) $$\Omega =\{\alpha_{1},\alpha_{2},\alpha_{3},\cdots ,\alpha_{N}\}$$

The power set space of FOD composed of *N* propositions composed of Ω is defined as

(2) $$2^{\Omega }=\left\{\emptyset ,\left\{\alpha_{1}\right\},\cdots ,\left\{\alpha_{1},\alpha_{2}\right\},\cdots ,\left\{\alpha_{1},\alpha_{2},\cdots ,\alpha_{N}\right\}\right\}$$

In the complete set of Ω, mass function (also known as BPA) is a mapping relation of $2^{\Omega }\to \left[0,1\right]$, and the classical D-S evidence theory satisfies the following relation

(3) $$\begin{array}{c} m(\emptyset )=0\\ \sum_{A\in 2^{\Omega }}m\left(A\right)=1 \end{array}$$

If A⊂Ω and m(A)>0, then *A* is called a focal element of the complete set. If A≠∅, the value of mass function m(A) indicates the support of evidence source for *A*; the larger the value of m(A), the more support for *A*.

In $2^{\Omega }\to \left[0,1\right]$, the belief function of a BPA is defined as

(4) $$Bel\left(A\right)=\sum_{B\subseteq A}m\left(B\right)$$

where *B* is not equal to an empty set. Bel(A) is a measure of the sure value of event *A*.

Assuming that there are two independent evidence sources, $m_{1}$ and $m_{2}$, and there is a certain conflict *k* between the two evidence sources, then *k* is defined as

(5) $$k=\sum_{B,C\in 2^{\Omega },A\cap B=\emptyset }m_{1}\left(B\right)m_{2}\left(C\right)$$

When the *k* value is 1, it means that the BPAs given by the two evidence sources are completely in conflict. When the value of *k* is 0, there is no conflict between them. Using the Dempster–Shafer evidence theory to combine them and using ⊕ to represent the operation of combination, the Dempster combination rule is as follows

(6) $$m\left(A\right)=m_{1}⊕m_{2}=\frac{\sum_{B,C\in 2^{\Omega },B\cap C=A}m_{1}\left(B\right)m_{2}\left(C\right)}{1-k}$$

where the value of *k* is not equal to 0.

At the same time, the above situation can be extended to multi-evidence source scenarios that are independent of each other. If there are *n* evidence sources, the combination process at this time is as follows

(7) $$m\left(A\right)=m_{1}⊕m_{2}⊕m_{3}⊕\cdots ⊕m_{n}$$

### 2.2. The Distance between Betting Commitments of the BPAs

Let *m* be a BPA in the FOD. Its associated pignistic probability function $BetP_{m}$ is defined on $2^{\Omega }\to \left[0,1\right]$ as [55]

(8) $$BetP_{m}\left(\lambda \right)=\sum_{A\subseteq \Omega ,\lambda \in A}\frac{1}{\left|A\right|}\frac{m(A)}{1-m(\emptyset )},\forall \lambda \in \Omega$$

where |A| represents the cardinality of set *A* and m(∅) is not 0. $BetP_{m}$ effectively measures the impact of a subset of set *A* on set *A* and strengthens the correlation between the factors of the complete set. Therefore, on $2^{\Omega }\to \left[0,1\right]$, the $BetP_{m}$ of the set *A* is defined as

(9) $$BetP_{m}\left(A\right)=\sum_{\lambda \in A}BetP_{m}\left(\lambda \right)$$

The transformation of mass function to $BetP_{m}$ is called pignistic transformation, which has been proven to be effective [55]. In the formula, if m(∅)=0, $BetP_{m}\left(\lambda \right)$ degenerates to

(10) $$BetP_{m}\left(\lambda \right)=\sum_{A\subseteq \Omega ,\lambda \in A}\frac{1}{\left|A\right|}m\left(A\right)$$

$BetP_{m}\left(A\right)$ strengthens the relationship between *A* sets and their subsets, and its significance lies in measuring the total quality value of *A*, which is called the betting commitment of *A*. In the following, we have always believed that $BetP_{m}$ has been extended to the collection $2^{\Omega }$.

Suppose two independent evidence sources, $m_{1}$ and $m_{2}$, belong to FOD Ω. $BetP_{m_{1}}$ and $BetP_{m_{2}}$ are the results of their pignistic transformation, respectively. Define the betting commitment distance between the two evidence sources as [27]

(11) $$difBetP_{m_{2}}^{m_{1}}=max_{A\in \Omega }(|BetP_{1}\left(A\right)-BetP_{2}\left(A\right)\left|\right)$$

where the value of $|BetP_{1}\left(A\right)-BetP_{2}\left(A\right)|$ represents the betting commitment difference value between all subsets of the two evidence sources. We select the largest of the difference values as the distance between different sources. For example, when the maximum subset betting commitment difference between the two sources is 1, it is proven that they completely conflict with each other. The maximum betting commitment difference between two sources that have no conflict at all is 0. Later, $difBetP_{m_{2}}^{m_{1}}$ is uniformly written as difBetP.

### 2.3. Management of Conflict Data

**Example 1.**  *Suppose the FOD is Ω={a,b,c}, the BPAs given by two sources of evidence are as follows* [26]:

$$\begin{array}{cc} \; & m_{1}\left(a\right)=0.99,m_{1}\left(b\right)=0.01\\ \; & m_{2}\left(b\right)=0.01,m_{2}\left(c\right)=0.99 \end{array}$$

*The conflict coefficient k=0.9999 is calculated, which reflects the obvious shortcomings of the classical Dempster theory.*

In the case of high conflict, low BPA fusion is amplified. In this combination, $m_{1}$ and $m_{2}$ hardly support {b}, but the fused m(b)=1. This can be concluded from the Dempster combination rule that $\frac{1}{1-k}$ makes the coefficient of high conflict fusion too large.

The absoluteness of the data leads to the irreversibility of the combination results [54]. If the mass function of a hypothesis is 0, then the hypothesis will always be 0 [48]. The combination rule requires that one of the two fused sources is 0 at the same time, and the result will not be 0. Evidence sources 1 and 2 strongly support {a} and {c}, respectively. {a} in source 2 is 0, resulting in the fused m(a)=0, similarly m(c)=0. The reason for anti-intuition lies in the fact that BPAs generated by directly using classical evidence theory and completely trusting several evidence sources by default, and the negation of one evidence source has a decisive impact on the overall situation.

The combination rule of the classical D-S evidence theory satisfies the law of exchange and association, etc. It is easy to lose these beautiful properties when modifying the rule. Considering the objectivity of the integration process, it is not a good idea to destroy these properties. For example, Yager’s improved method [33] allocates all the conflict parts to the unknown, and the synthesis result belongs to a conservative strategy, which enhances the uncertainty of the proposition. In particular, when there is a large amount of evidence, this method cannot help to provide decision-making services. This affects the ability of the D-S evidence theory to be popularized and applied in real life.

Therefore, it is a reasonable method to carry out appropriate preprocessing on the data to adapt to the combination rule.

#### 2.3.1. Murphy’s Method: Average Values of BPA

Murphy proposed a method to modify the original evidence [48]. First, the arithmetic average of BPA values of all the evidence is obtained, and then the data are fused by the classical combination rule. Its advantage is avoiding the problem that a certain source BPA is incomplete and cannot be integrated. Although this method is effective for the final result, a lot of information is lost in the process. If there is a strong conflict between the two sources, the averaging operation can strongly change the support level of the sources. This has actually weakened a lot of information in the evidence. The data used in the fusion cannot represent conflicts in the evidence. At the same time, if the same BPA for all sources is zero, the fusion result is still 0. According to the correlation between factors in the total set, even if the current evidence thinks that the result is 0, a certain BPA value should be given to achieve the expected application effect.

#### 2.3.2. Wang et al. Method: Conflict Management with Base Belief Function

Wang et al. proposed a solution for conflict management [54]. Specifically, assuming that there are elements in the FOD, the base belief function is defined as

(12) $$\bar{m}\left(A_{i}\right)=\frac{1}{2^{N}-1}$$

where $A_{i}$ is a subset of the total set and is non-empty.

The BPAs of the basic belief function and the evidence source are weighted and averaged to obtain new evidence

(13) $$m^{\prime }\left(A_{i}\right)=\frac{\bar{m}\left(A_{i}\right)+m\left(A_{i}\right)}{2}$$

$m^{\prime }\left(A_{i}\right)$ is the new value of evidence for the combination rule. Before every new source appears, the belief in every situation must be equal. Give the original possibility to ensure its existence and give each BPA a certain fault tolerance rate. The advantage of this method for processing the original data is that it maintains the basic characteristics of the original data and eliminates the complete contradiction between the evidence. Further, it satisfies the exchangeability and association.

Although the amount of evidence can continue to increase, it is not certain that a certain piece of evidence is right. The uncertainty of right and wrong evidence does not have a reasonable measurement method in this method. It is reckless to weigh only the basic belief function and BPA with equal weight.

## 3. A New Evidence Reliability Coefficient

In this part, the concepts of the single factor belief function and a new evidence reliability coefficient are proposed. According to the reliability coefficient, the original evidence and the single factor belief function are weighted to obtain an effective conflict management solution.

### 3.1. The Proposed Measure

Suppose, in a closed world, *N* elements are included in the FOD Ω. $2^{\Omega }$ is the power set of Ω, in which there are $2^{N}$ elements. The single factor belief function $m_{s}\left(A_{i}\right)$ is defined as

(14) $$\begin{array}{cc} m_{s}\left(A_{i}\right) & =\frac{1}{\sum_{i\in 2^{N}-1}\left|A_{i}\right|}=\frac{1}{N+2C_{N}^{2}+3C_{N}^{3}+\cdots +NC_{N}^{N}} \end{array}$$

where $A_{i}$ represents the i−th non-empty subset in the complete set, and $|A_{i}|$ represents the potential of the subset $A_{i}$. The single factor belief function is the reciprocal of the sum of the potentials of all non-empty subsets in the total set. Equations (12) and (14) have the same point in that they rely on the existence of a closed FOD.

The evidence reliability coefficient ε is defined as

(15) $$\varepsilon =1-\frac{difBetP}{2}$$

where difBetP is the largest betting commitment difference between the evidence sources defined in Equation (11).

Then the original BPA value is processed by using single factor belief function and evidence reliability coefficient. Let the treated BPA value be $m^{\prime \prime }\left(A_{i}\right)$, and *i* is the number of BPA. Then, the conflict coefficient *k* is calculated for fusion. m(A) represents the fused BPA. Then, use the two-dimensional form of 〈ε,m(A)〉 to represent the fused results. m(∅)=0, weighted by the evidence reliability coefficient, $m^{\prime \prime }\left(A_{i}\right)$ is defined as

(16) $$m^{\prime \prime }\left(A_{i}\right)=\left(1-\varepsilon \right)\left|A_{i}\right|m_{s}\left(A_{i}\right)+\varepsilon m\left(A_{i}\right).$$

The purpose of using the single factor belief function is to make up for the defects in the combination rule itself. The single factor belief function is a single subset that evenly distributes the probability to each subset, while the basic belief function in Equation (12) evenly distributes the probability to each subset of the total set. The purpose of the basic belief function is to give each subset in the FOD an equal possibility before generating BPA [54]. Assuming that every subset of Ω has BPA, the subset with small potential has more combination items than the subset with large potential in the fusion process, which may lead to the small fusion result of BPA with large potential in the source evidence. We call this phenomenon fusion bias. Therefore, it is more reasonable to use the single factor belief function to compensate the subset with larger potential in the reliability weighting rule, which is more reasonable than the average distribution of the basic belief function. The basic belief function and the original evidence are weighted by 0.5, and the fusion bias of the classical Dempster combination rule is not fully considered.

Using the evidence reliability coefficient to weaken the proportion of original evidence in fusion. If there is no difference between evidence sources, then the combination rule can completely trust evidence, and the system degenerates into the classical D-S evidence theory. The reliability of the evidence here is not less than 0.5 to avoid excessive preprocessing of the evidence. If the evidence does not have any belief, the system will assign all the single factor belief functions to BPAs, and the combination will lose its meaning. 〈ε,m(A)〉 tells the user the reliability of the combined BPAs. The reliability of evidence is closely related to the evidence itself. It changes with the change in evidence and measures the reliability of a given set of evidence. When the amount of evidence is sufficient, a relatively stable betting commitment difference range is screened, and a belief degree is fixed to avoid the huge impact of extreme data on the whole.

The advantage of the reliability coefficient is that it not only eliminates the complete contradiction between evidence but also adjusts the proportion of the single factor belief function in fused data. The reconciliation of reliability makes the data used in fusion more reasonable. The occurrence of high-conflict situations means that the reliability of evidence is reduced, and it is impossible to know in advance which source of evidence is correct. The system remains skeptical of all events unless there is only one source of evidence. The combination result itself is the probability guess value of the event occurrence, not the accurate data obtained by the sensor. Therefore, the inaccuracy of the data source will not have a decisive impact on the final result [54]. In given evidence sources, *A* and *B*, if they give a very low BPA value to an event at the same time that the belief degree is low, the modified BPA value of the proposition will become a bigger value due to the weight effect, and it will become a bigger value after combination. The proposed measure method can solve this problem.

There are many measures for uncertainty management in evidence theory based on different elements of evidence [34,36,37]. The evidence element in the proposed measure is compared with some classical and typical uncertainty measures in Table 1. It can be seen that the evidence elements *m*, Pl, Bel, $\left|A\right|$ and $BetP_{m}$ are commonly used.

### 3.2. Numerical Examples of Using New Evidence Reliability Coefficient

Some numerical examples are given as followsm and more numerical examples of the new evidence reliability coefficient can be found in the previous conference work in [66].

**Example 2.**  *Suppose the FOD is Ω={a,b,c,d}, and the BPAs given by the two evidence sources are*

$$\begin{array}{ccc} \; & \; & m_{1}\left(a,b\right)=1.00,m_{1}\left(c,d\right)=0.00\\ \; & \; & m_{2}\left(a,b\right)=0.00,m_{2}\left(c,d\right)=1.00 \end{array}$$

*First, the single factor belief function of this FOD is obtained from the formula*

(17) $$\begin{array}{cc} m_{s}\left(A_{i}\right) & =\frac{1}{1\times C_{4}^{1}+2\times C_{4}^{2}+3\times C_{4}^{3}+4\times C_{4}^{4}}=\frac{1}{32} \end{array}$$

*Then, the reliability coefficient for this group of evidence is calculated as ε=0.5. The evidence function of the modified $m_{1}$ is shown in Table 2.*

**Figure 1:**
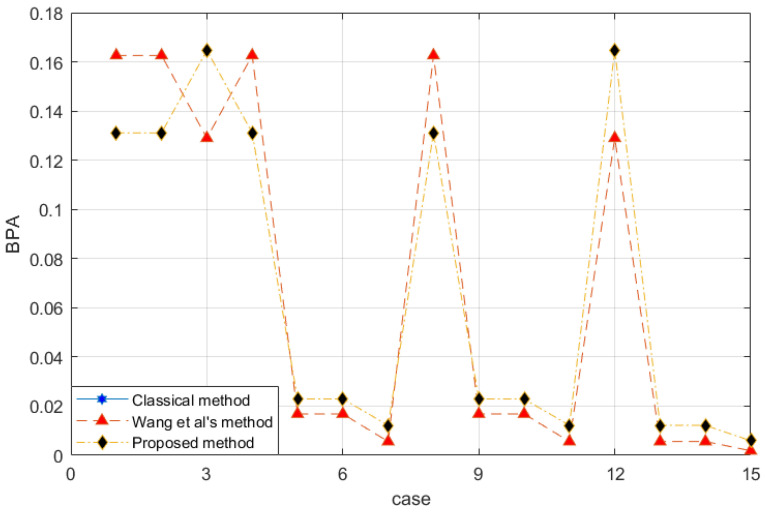
Evidence fusion result of Example 2.

The modified data of $m_{2}$ is similar to that of $m_{1}$, except $m_{2}\left(a,b\right)=0.0313$ and $m_{2}\left(c,d\right)=0.5313$. We recalculate the conflict coefficient to be k=0.3389. The combination rule is introduced and compared with the classical D-S evidence theory, the basic belief function proposed by Wang et al. [54]. Figure 1 is the fusion result.

The horizontal axis of Figure 1 is arranged in the binary order of {d,c,b,a} and represents the mass function of the corresponding case. For example, binary 1 represents {a}, and binary 1011 represents {d,b,a}, or {a,b,d}.

According to the classical D-S evidence theory, the conflict value of the two evidence sources is 1 and cannot be combined. Therefore, the blue line is not shown in the figure. This set of evidence is completely conflicting. Wang et al.’s method [54] and the proposed method both disperse the information contained in the evidence into related subsets. The results show that in Wang et al.’s method [54], m(a,b) is less than m(a) and m(b), and m(c,d) is less than m(c) and m(d), which is unreasonable. Because the evidence does not directly tell the system that the BPA of the single subset is larger. The proposed method improves the defects in this aspect.

**Example 3.**  *Suppose the FOD is Ω={a,b,c,d}, and the BPAs given by the two evidence sources are* [27]:

$$\begin{array}{cc} \; & m_{1}\left(a,b,d\right)=0.80,m_{1}\left(c\right)=0.10,m_{1}\left(d\right)=0.10\\ \; & m_{2}\left(a,b\right)=0.10,m_{2}\left(c\right)=0.10,m_{2}\left(d\right)=0.80 \end{array}$$

**Figure 2:**
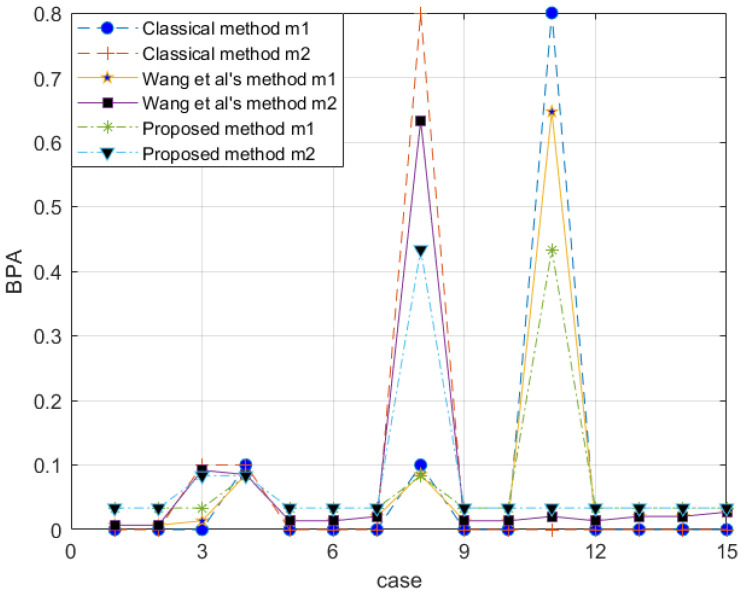
Comparison of evidence preprocessing result in Example 3.

**Figure 3:**
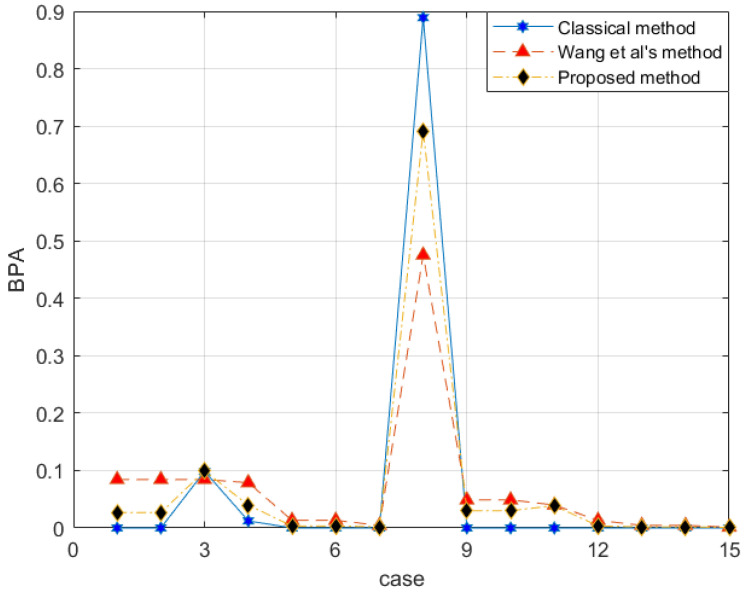
Evidence fusion result of Example 3.

*With the proposed method, the evidence after preprocessing with different methods is shown in Figure 2, and the fusion result is shown in Figure 3.*

The reliability coefficient of the two pieces of evidence is ε=0.7835. After observing the characteristics of the data in Figure 3 and modifying the evidence, the proposed method better retains the characteristics of the original data. Wang et al.’s method uses 0.5 weight to excessively modify the evidence. Especially when the FOD is small, if the original BPA value is small, the modified evidence will be too large, resulting in large errors. In the combined results, the m(a,b) of all methods is almost the same. Regarding m(d), Wang et al.’s method is too conservative. The results produced by the proposed method are between the classical theory and Wang et al.’s method, which is also more reasonable. For m(c), the proposed method appropriately distributes the probability in other subsets and is available within the error range.

## 4. Application

**Figure 4:**
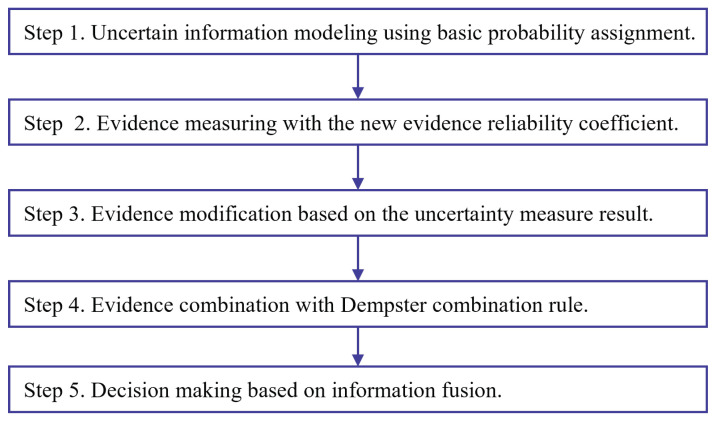
The flow chart of uncertain information fusion based on the new evidence reliability coefficient.

To evaluate the effectiveness of the proposed uncertainty measure, a new method of uncertain information fusion based on the new evidence reliability coefficient is proposed in Figure 4. With the proposed method, two UCI data sets are adopted for experiment and verification.

The steps of uncertain information fusion in Figure 4 with the new evidence reliability coefficient are demonstrated as follows.

Step 1 Uncertain information modeling using basic probability assignment.
  Uncertain information modeling using basic probability assignment is the first step of applying D-S evidence theory. There are many methods for BPA generation [67].
Step 2 Evidence measuring with the new evidence reliability coefficient.
  The single factor belief function of this FOD is calculated based on Equation (14), and the evidence reliability coefficient ε is calculated based on Equation (15).
Step 3 Evidence modification based on the uncertainty measure result.
  After evidence measuring with the new evidence reliability coefficient in Equation (15), the original evidence can be modified based on Equation (16).
Step 4 Evidence combination with Dempster combination rule.
  The Dempster combination rule is applied for evidence fusion after evidence modification.
Step 5 Decision-making based on information fusion.
  For practical applications such as classification and identification, the decision-making can be made after information fusion steps.

### 4.1. Experiment 1

The first data set is the banknote authentication data set [68] from the machine learning repository UCI [69]. The data were extracted from pictures taken of real and forged banknote specimens. Each image has a pixel size of 400×400, obtaining a gray image with a resolution of about 600 dpi. Wavelet transform tools are used to extract features from images. The four characteristics are: variance of the image after wavelet transform, skewness of the image after wavelet transform, curvature of the image after wavelet transform and image entropy. The above characteristics are all values under continuous transformation. The fifth dimension represents sample classification, where 0 represents genuine banknotes, and 1 represents counterfeit banknotes. There were 610 and 773 cases of genuine and counterfeit banknotes, respectively. We select their last 10 test cases. The triangular fuzzy numbers [70] of the four characteristics of real and counterfeit banknotes are generated by using the remaining samples, such as Table 3.

In the example, each feature is equivalent to an independent evidence source, and these evidence sources are independent of each other. Then the proposed method is applied to the test set. We select test cases (−1.3887,−4.8773,6.4774,0.34179,0). After modifying the evidence, it is brought into the combination rule to obtain the result of Table 4. In the original evidence, m(a) indicates real banknotes and m(b) indicates counterfeit banknotes. m(a,b) has no practical meaning. That is, the uncertainty in the system calculation process.

**Figure 5:**
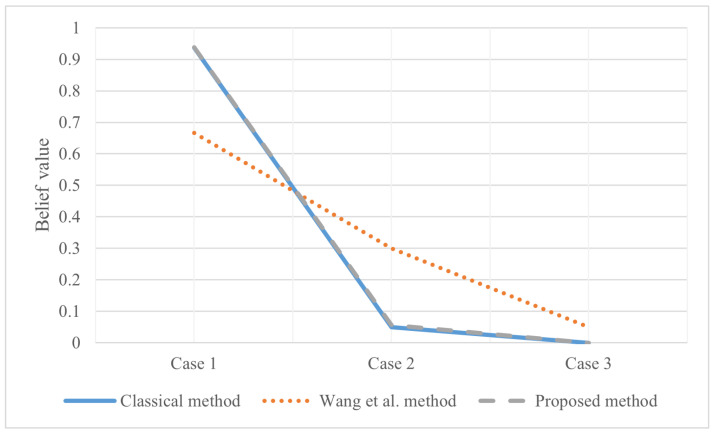
The evidence combination result of Experiment 1.

After evidence combination, the results shown in Figure 5 are obtained. According to Figure 5, the decision-making conclusion of the proposed method is 〈0.8031,0.9367〉; that is, judging the category according to these four characteristics, it is considered that the sample has an assurance of 0.9367 as the real banknote. At the same time, the data measured by these four characteristics have a probability of 0.8031 to be trusted. Of course, the labels in the data set are also real banknotes. It proved to be very effective. The classical D-S evidence theory also performs well in this example. However, Wang et al.’s method [54] is less impressive. At the same time, it can be seen that Wang et al.’s method increases the uncertainty of the system itself in the face of the problem that only a single subset BPA is meaningful.

### 4.2. Experiment 2

The second dataset is still adopted from UCI. This data set is the Iris data, which is frequently used in classification experiments [71]. This is a case of linear separability. Among them, there are four attributes, namely sepal length, sepal width, petal length and petal width; they are all measured in centimeters. A total of 150 samples were divided into 3 categories on average: Setosa, Versicolour and Virginica. They are represented by the letters *a*, *b* and *c*, respectively. A total of 45 samples are randomly selected from each category to construct the corresponding triangular fuzzy number. Table 5 gives an example after selection. Intuitively, there are significant differences in the values for triangular fuzzy numbers models on different attributes in the sample.

**Figure 6:**
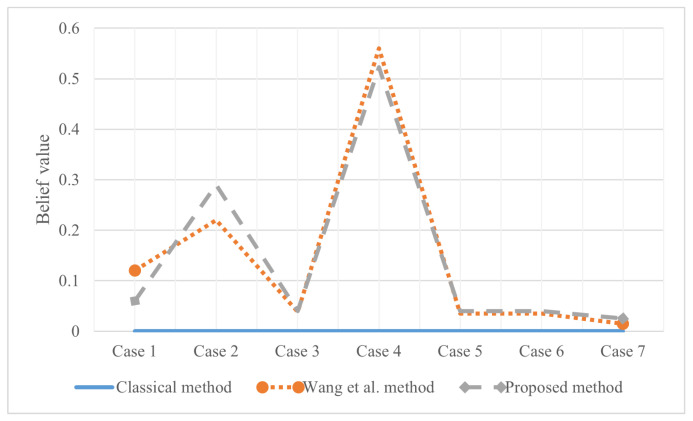
The evidence combination result of Experiment 2.

The classical combination rule in D-S evidence theory is easy to show extreme errors when the subset of a single factor is incomplete and non-zero. Samples (6.1,2.6,5.6,1.4,2) are extracted from the test set. A set of pieces of evidence in Table 6 is obtained by using single factor belief function. It is verified by other examples that the three fusion methods can obtain interpretable BPA values. However, in this example, because the mass function of different iris varieties is determined to be 1 by the two evidence sources, respectively, the classical method fails to give the result, and the variety is considered to be outside the FOD. Wang et al.’s method [54] is also satisfactory. According to Figure 6, the combination results obtained are 〈0.8107,0.5226〉, which means that the sample has a belief of 0.5226 of the Virginica variety. Further, the reliability of the evidence sources is 0.8107. The side also verifies that the proposed method will have higher accuracy on similar data sets.

### 4.3. Analysis of Application Result

According to the experimental results in Experiments 1 and 2, the proposed method is robust and can be adopted in extreme situations with uncertain data environments. If there are only mono-subsets, the proposed method weakly maintains the possibility of non-mono-subsets, which is caused by the intersection zone among different characteristics in Experiment 1 and different properties in Experiment 2 in the sample. It is also a kind of uncertainty. In this way, other values in the combined result can be explained. Take Experiment 2 as an example, the Iris varieties in the case are judged according to their attributes. If the division effect of a certain attribute is obvious, then the more obvious the role it plays in the combination process. The probability of dividing the same sample by different attributes is quite different, which fully proves that the evidence is unreliable or the attributes need pruning. Then the evidence theory can be used as a good method for decision pruning. Adding a certain attribute, the classification result is strengthened or changed, proving that the attribute can be used for classification. On the contrary, it needs to be abandoned. In practical application, if there are too many attributes in the data set, several attributes can be removed in advance to reduce the computation and increase the robustness of the system.

In the proposed method, the new evidence reliability coefficient and the single factor belief function are used simultaneously for uncertainty management in the source of evidence. Due to the existence of the single factor belief function, the elements in the whole FOD can be addressed as a complete information source. No matter how high the conflict between different pieces of evidence is, it will be reduced by the redistribution of BPA values after evidence modification with the evidence reliability coefficient. It should be noted that the fused BPA obtained after information fusion is a probability trend. If the source number of evidence is far more than the number of subsets of the FOD, the statistical results are not only closer to the actual situation, but also the cost is almost the lowest. The proposed conflict data management method keeps the mathematical characteristics in the Dempster combination rule without adding new computation complexity compared to other evidence preprocessing methods. The computational complexity of the proposed method as an integrated data fusion process can be denoted as $O=2^{N}$, where *N* is the number of elements in the FOD. This is because the calculation of Equation (5) in the Dempster combination rule needs to traverse all the cases’ occurrence probability of the evidence source in the FOD while other calculations can be reduced. The proposed method adds no new computational complexity.

## 5. Discussion and Open Issue

If the Dempster–Shafer evidence theory is used to deal with conflict data directly, it is likely that the combination results are counter-intuitive due to data defects. The classical Dempster combination rule has good exchangeability and combination while destroying combination structures often leads to different results. Appropriate modifications to the data model can improve the performance of the combination rule. The method proposed in this paper defines the one-factor belief function on the basis of the closed FOD, which not only makes the mass function of each subset non-zero, it avoids the combination defects of the classical D-S evidence theory and also balances the preference degree for the single factor set in the combination process to a certain extent. Small subsets always have more intersections than large subsets. The proposed factor 〈ε,m(A)〉, a two-dimensional evaluation form, can give a more intuitive reference to the outside world. The conflict coefficient in the classical combination rule cannot better reflect the difference between evidence. The new evidence reliability coefficient is defined based on the maximum betting commitment distance between evidences. The reliability is low if the distance is large. For evidence with high reliability, the proposed method can better maintain the characteristics of the original data. In addition, when the mass function of a single subset or a complete set is not zero, the classical D-S evidence theory can perform well. In order to reduce the computational complexity, new methods can be abandoned in this case. The defined one-factor belief function is only applicable to the closed world. At the same time, the maximum betting commitment distance is under the closed conditions and m(∅)=0. If sensors are used to collect data, the FOD may gradually become larger during use, and the relative strain of the single factor belief function is small. When the FOD is small, the combination has a greater fault tolerance, which reduces the accuracy of the system. Larger FOD, more evidence and single factor belief function reduce the influence on the original data, improving the accuracy of probability. The classical D-S evidence theory itself has exponential disasters. The new method requires traversing every subset of the FOD in computation, but only increases the computation by a low multiple. They were reduced to be equivalent.

There are two ways to explain belief functions [72]. One is to treat BPAs as generalized probabilities, and the other is to treat them as a way to express evidence. From the first point of view, the influence of single factor belief function on a non-single factor subset is considered. If the number of intersections caused by the potential of the subset is small, it is equivalent to directly weakening the combination probability of this term. In other words, non-single factor subsets bear more conflicts but cannot express them. Specifically, for the two evidence sources $m_{1}$ and $m_{2}$, if there is a complete set of evidence BPA that is not zero, then it can match any focal element. The fusion process is greatly limited, and the complete set BPA of another source must also be non-zero. It is reasonable to allocate part of the probability back to these subsets. The weighting of the single factor belief function according to the reliability coefficient is also afraid of excessive damage to the original data structure. The proposed method is not only to make the results of the classical Dempster combination rule more reasonable but also to improve some irreconcilable contradictions between evidence. For example, the combination of zero and non-zero for the same case. Under the condition of ensuring the integrity of the evidence source (such as sensors), BPA with very low reliability is still obtained. It is possible to sort out the uncertainty of this matter to the environment rather than the source. Of course, outliers always appear, and they have a great influence on the proposed method. If there are enough sources or they appear in real-time, a minimum degree of trust can be specified to exclude evidence that causes low reliability. It not only reduces the amount of calculation but also reduces the contradictions of the system and makes the combination results more credible.

There are some open issues in the proposed measure that need to be addressed in the following research. First, the proposed reliability coefficient is based on the betting commitment evidence distance in the Dempster–Shafer evidence theory. However, the pignistic transformation of a BPA in the betting commitment evidence distance may have some undesirable properties, as shown in [73]. Take monotonicity as an example, the pignistic transformation of a BPA can express an increase in information when the real situation is on the contrary. Second, the mathematical characteristics of the proposed reliability coefficient can be taken into consideration if it is not regarded as a simple factor for evidence modification. These properties include probabilistic consistency, set consistency, range, subadditivity, additivity and monotonicity. It should be noted that the property of a measure in the Dempster–Shafer evidence theory is a very big problem as well as an open issue that attracts a lot of attention among studies [64,65,74,75]. Third, there may be problems that occur in the application of the new measure to other data sets. An example is the dependence on evidence. If the dependence of the sources of evidence is not guaranteed, then a new evidence combination rule may be needed. Another concern may focus on incomplete FOD, where there are different FODs during information fusion. Then, the generalized evidence theory [28,30] may be a choice and direction.

## 6. Conclusions

A new reliability coefficient using betting commitment evidence distance and the single factor belief function in the Dempster–Shafer evidence theory for conflict and uncertain information fusion is proposed in this work. Evidence preprocessing is based on the new reliability coefficient and single factor belief function. The merit of the evidence preprocessing process is that it takes advantage of the characteristics of the evidence itself to deal with the uncertain data and avoids the problem that the classical Dempster combination rule is not applicable for high-conflict evidence. After evidence modification, the Dempster combination rule is used for information fusion. A new method of uncertain information fusion is proposed based on the new measure. Experiments on two UCI data sets are designed to verify the rationality and effectiveness of the proposed method. The following work can focus on (1) applying the proposed method in other engineering fields and (2) addressing the open issues in uncertainty measures of the evidence theory, such as the properties of a measure.

## Figures and Tables

**Table 1:** Uncertainty measures in the Dempster–Shafer evidence theory.

Uncertainty Measure	Definition	Evidence Element
Hohle’s confusion measure [56]	$C_{H}\left(m\right)=-\sum_{A\subseteq X}m\left(A\right)\log_{2}Bel\left(A\right)$	*m*, Bel
Yager’s dissonance measure [57]	$E_{Y}\left(m\right)=-\sum_{A\subseteq X}m\left(A\right)\log_{2}Pl\left(A\right)$	*m*, Pl
Dubois and Prade’s weighted Hartley entropy [58]	$E_{DP}\left(m\right)=\sum_{A\subseteq X}m\left(A\right)\log_{2}\left|A\right|$	*m*, $\left|A\right|$
Klir and Ramer’s discord measure [59]	$D_{KR}\left(m\right)=-\sum_{A\subseteq X}m\left(A\right)\log_{2}\sum_{B\subseteq X}m\left(B\right)\frac{\left|A\cap B\right|}{\left|B\right|}$	*m*, $\left|A\right|$
Klir and Parviz’s strife measure [60]	$S_{KP}\left(m\right)=-\sum_{A\subseteq X}m\left(A\right)\log_{2}\sum_{B\subseteq X}m\left(B\right)\frac{\left|A\cap B\right|}{\left|A\right|}$	*m*, $\left|A\right|$
George and Pal’s total conflict measure [61]	$TC_{GP}\left(m\right)=\sum_{A\subseteq X}m\left(A\right)\sum_{B\subseteq X}m\left(B\right)\left(1-\frac{\left|A\cap B\right|}{\left|A\cup B\right|}\right)$	*m*, $\left|A\right|$
Jousselme et al. measure [62]	$AM\left(m\right)=-\sum_{x\in X}BetP_{m}\left(x\right)\log_{2}\left(BetP_{m}\left(x\right)\right)$	*m*, $BetP_{m}$
Deng entropy [63]	$E_{d}\left(m\right)=-\sum_{A\subseteq X}m\left(A\right)\log_{2}\frac{m\left(A\right)}{2^{\left|A\right|}-1}$	*m*, $\left|A\right|$
Jirousek et al. measure [64]	$H\left(m\right)=H_{s}\left(Pl_{-}P_{m}\right)+H_{d}\left(m\right)$	*m*, Pl
Pan et al. measure [65]	$H_{bel}\left(m\right)=-\sum_{A\subseteq 2^{X}}\frac{Bel\left(A\right)+Pl\left(A\right)}{2}\log_{2}\frac{Bel\left(A\right)+Pl\left(A\right)}{2\left(2^{\left|A\right|}-1\right)}$	*m*, Bel, Pl
Proposed measure	$\varepsilon =1-\frac{difBetP}{2}$	*m*, $BetP_{m}$

**Table 2:** The results of the $m_{1}$ preprocessing of Example 2.

**Evidence**	$m_{1}\left(a\right)$	$m_{1}\left(b\right)$	$m_{1}\left(c\right)$	$m_{1}\left(d\right)$	$m_{1}\left(a,b\right)$	$m_{1}\left(a,c\right)$	$m_{1}\left(a,d\right)$	$m_{1}\left(b,c\right)$
$m^{\prime \prime }\left(A_{i}\right)$	0.0156	0.0156	0.0156	0.0156	0.5313	0.0313	0.0313	0.3130
**Evidence**	$m_{1}\left(b,d\right)$	$m_{1}\left(c,d\right)$	$m_{1}\left(a,b,c\right)$	$m_{1}\left(a,b,d\right)$	$m_{1}\left(a,c,d\right)$	$m_{1}\left(b,c,d\right)$	$m_{1}\left(a,b,c,d\right)$	
$m^{\prime \prime }\left(A_{i}\right)$	0.0313	0.0313	0.0469	0.0469	0.0469	0.0469	0.0625	

**Table 3:** Triangular Fuzzy Numbers with the Four Characteristics of Experiment 1.

Attribute	Real Banknotes	Counterfeit Banknotes
Variance	(−7.0421, −1.7931, 2.3917)	(−4.2859, 2.5531, 6.8248)
Skewness	(−13.7731, 0.1821, 9.6014)	(−6.9321, 5.6688, 12.9516)
Curvature	(−5.2861, 0.2752, 17.9274)	(−4.9417, 0.7006, 8.8294)
Image Entropy	(−7.5887, −0.6629, 2.1353)	(−8.5482, −0.5524, 2.4495)

**Table 4:** Test Case Evidence Processing of Experiment 1.

	Varience	Skewness	Curvature	Image Entropy
m(a)	0.6607	0.7696	0.6606	0.4474
m(b)	0.3200	0.2112	0.3106	0.4835
m(a,b)	0.0385	0.0385	0.0575	0.1382

**Table 5:** Triangular Fuzzy Numbers with Four Characteristics of Experiment 2.

Attribute	Setosa	Versicolour	Virginica
Sepal length	(4.3, 5.0, 5.8)	(4.9, 6.0, 7.0)	(4.9, 6.5, 7.9)
Sepal width	(2.3, 3.4, 4.4)	(2.0, 2.8, 3.4)	(2.2, 3.0, 3.8)
Petal length	(1.0, 1.5, 1.9)	(3.3, 4.4, 5.1)	(4.5, 5.6, 6.9)
Petal width	(0.1, 0.2, 0.6)	(1.0, 1.3, 1.8)	(1.4, 2.0, 2.5)

**Table 6:** Test Case Evidence Processing of Experiment 2.

Attribute	m(a)	m(b)	m(a,b)	m(c)	m(a,c)	m(b,c)	m(a,b,c)
Sepal length	0.0146	0.4643	0.0293	0.3893	0.0293	0.0293	0.0439
Sepal width	0.1548	0.3887	0.0423	0.2662	0.0423	0.0423	0.0634
Petal length	0.0343	0.0343	0.0687	0.6223	0.0687	0.0687	0.1030
Petal width	0.0211	0.7674	0.0423	0.0211	0.0423	0.0423	0.0634

## Data Availability

All data generated or analysed during this study are included in this published article.

## References

[1] Jiroušek R., Shenoy P.P. (2020). On properties of a new decomposable entropy of Dempster-Shafer belief functions. Int. J. Approx. Reason..

[2] Song Q., Ni Y., Ralescu D.A. (2020). The impact of lead-time uncertainty in product configuration. Int. J. Prod. Res..

[3] Zhou T., Zhang X., Droguett E.L., Mosleh A. (2023). A generic physics-informed neural network-based framework for reliability assessment of multi-state systems. Reliab. Eng. Syst. Saf..

[4] Zhang X., Mahadevan S. (2019). Ensemble machine learning models for aviation incident risk prediction. Decis. Support Syst..

[5] Liu Z.g., Zhang Z., Liu Y., Dezert J., Pan Q. (2019). A new pattern classification improvement method with local quality matrix based on K-NN. Knowl.-Based Syst..

[6] Kang B., Chhipi-Shrestha G., Deng Y., Hewage K., Sadiq R. (2018). Stable strategies analysis based on the utility of Z-number in the evolutionary games. Appl. Math. Comput..

[7] Ho W., Ma X. (2018). The state-of-the-art integrations and applications of the analytic hierarchy process. Eur. J. Oper. Res..

[8] Yang X., Ni Y. (2020). Size Relation of Uncertain Sets with Application to Clustering. J. Intell. Fuzzy Syst..

[9] Fu C., Chang W., Xue M., Yang S. (2019). Multiple criteria group decision making with belief distributions and distributed preference relations. Eur. J. Oper. Res..

[10] Deng X., Jiang W. (2018). An evidential axiomatic design approach for decision making using the evaluation of belief structure satisfaction to uncertain target values. Int. J. Intell. Syst..

[11] Li H., Pan D. (2017). Multi-photoelectric detection sensor target information recognition method based on DS data fusion. Sensors Actuators A Phys..

[12] Xu X., Weng X., Xu D., Xu H., Hu Y., Li J. (2020). Evidence updating with static and dynamical performance analyses for industrial alarm system design. ISA Trans..

[13] Wu G.Q., Li L., Li L., Wu X. (2016). Web news extraction via tag path feature fusion using ds theory. J. Comput. Sci. Technol..

[14] Liu Z.G., Liu Y., Dezert J., Cuzzolin F. (2020). Evidence Combination Based on Credal Belief Redistribution for Pattern Classification. IEEE Trans. Fuzzy Syst..

[15] Liu Z.G., Huang L.Q., Zhou K., Denoeux T. (2020). Combination of transferable classification with multisource domain adaptation based on evidential reasoning. IEEE Trans. Neural Netw. Learn. Syst..

[16] Jiao L., Wang F., Liu Z.G., Pan Q. (2022). TECM: Transfer learning-based evidential c-means clustering. Knowl.-Based Syst..

[17] Zhou K., Martin A., Pan Q., Liu Z. (2018). SELP: Semi–supervised evidential label propagation algorithm for graph data clustering. Int. J. Approx. Reason..

[18] Su Z.g., Denoeux T. (2018). BPEC: Belief-peaks evidential clustering. IEEE Trans. Fuzzy Syst..

[19] Jiao L., Yang H., Liu Z.G., Pan Q. (2022). Interpretable fuzzy clustering using unsupervised fuzzy decision trees. Inf. Sci..

[20] Song Y., Zhu J., Lei L., Wang X. (2020). Self-adaptive combination method for temporal evidence based on negotiation strategy. Sci. China Inf. Sci..

[21] Tang Y., Tan S., Zhou D. (2022). An Improved Failure Mode and Effects Analysis Method Using Belief Jensen–Shannon Divergence and Entropy Measure in the Evidence Theory. Arab. J. Sci. Eng..

[22] Razi S., Mollaei M.R.K., Ghasemi J. (2019). A novel method for classification of BCI multi-class motor imagery task based on Dempster–Shafer theory. Inf. Sci..

[23] Zhao K., Li L., Chen Z., Sun R., Yuan G., Li J. (2022). A survey: Optimization and applications of evidence fusion algorithm based on Dempster-Shafer theory. Appl. Soft Comput..

[24] Ma W., Jiang Y., Luo X. (2019). A flexible rule for evidential combination in Dempster–Shafer theory of evidence. Appl. Soft Comput..

[25] Liu X., Liu S., Xiang J., Sun R. (2023). A conflict evidence fusion method based on the composite discount factor and the game theory. Inf. Fusion.

[26] Zadeh L.A. (1986). A simple view of the Dempster-Shafer theory of evidence and its implication for the rule of combination. AI Mag..

[27] Liu W. (2006). Analyzing the degree of conflict among belief functions. Artif. Intell..

[28] Deng Y. (2015). Generalized evidence theory. Appl. Intell..

[29] An J., Hu M., Fu L., Zhan J. (2019). A novel fuzzy approach for combining uncertain conflict evidences in the Dempster-Shafer theory. IEEE Access.

[30] Jiang W., Zhan J. (2017). A modified combination rule in generalized evidence theory. Appl. Intell..

[31] Yuan K., Deng Y. (2019). Conflict evidence management in fault diagnosis. Int. J. Mach. Learn. Cybern..

[32] Wang J., Qiao K., Zhang Z. (2019). An improvement for combination rule in evidence theory. Future Gener. Comput. Syst..

[33] Yager R.R. (1987). On the Dempster-Shafer framework and new combination rules. Inf. Sci..

[34] Jiang W. (2018). A correlation coefficient for belief functions. Int. J. Approx. Reason..

[35] Jiang W., Huang C., Deng X. (2019). A new probability transformation method based on a correlation coefficient of belief functions. Int. J. Intell. Syst..

[36] Zhou Q., Deng Y. (2022). Fractal-based belief entropy. Inf. Sci..

[37] Deng Y. (2020). Uncertainty measure in evidence theory. Sci. China Inf. Sci..

[38] Xiao F. (2019). Multi-sensor data fusion based on the belief divergence measure of evidences and the belief entropy. Inf. Fusion.

[39] Jiang W., Xie C., Zhuang M., Tang Y. (2017). Failure mode and effects analysis based on a novel fuzzy evidential method. Appl. Soft Comput..

[40] Chen L., Deng Y. (2018). A new failure mode and effects analysis model using Dempster–Shafer evidence theory and grey relational projection method. Eng. Appl. Artif. Intell..

[41] Denoeux T. (2019). Logistic regression, neural networks and Dempster–Shafer theory: A new perspective. Knowl.-Based Syst..

[42] Song Y., Wang X., Wu W., Quan W., Huang W. (2018). Evidence combination based on credibility and non-specificity. Pattern Anal. Appl..

[43] Zhang X., Mahadevan S., Deng X. (2017). Reliability analysis with linguistic data: An evidential network approach. Reliab. Eng. Syst. Saf..

[44] Sun Z., Zhang Z., Xiao C., Qu G. (2018). DS evidence theory based trust ant colony routing in WSN. China Commun..

[45] Xu X., Zhang D., Bai Y., Chang L., Li J. (2020). Evidence reasoning rule-based classifier with uncertainty quantification. Inf. Sci..

[46] Fu C., Hou B., Chang W., Feng N., Yang S. (2020). Comparison of Evidential Reasoning Algorithm with Linear Combination in Decision Making. Int. J. Fuzzy Syst..

[47] Tang Y., Chen Y., Zhou D. (2022). Measuring Uncertainty in the Negation Evidence for Multi-Source Information Fusion. Entropy.

[48] Murphy C.K. (2000). Combining belief functions when evidence conflicts. Decis. Support Syst..

[49] Dempster A.P. (2008). Upper and Lower Probabilities Induced by a Multivalued Mapping. Class. Work. -Dempster-Shafer Theory Belief Funct..

[50] Su X., Li L., Qian H., Mahadevan S., Deng Y. (2019). A new rule to combine dependent bodies of evidence. Soft Comput..

[51] Dusia A., Sethi A.S. (2016). Recent advances in fault localization in computer networks. IEEE Commun. Surv. Tutor..

[52] Chowdhuri I., Pal S.C., Chakrabortty R. (2020). Flood susceptibility mapping by ensemble evidential belief function and binomial logistic regression model on river basin of eastern India. Adv. Space Res..

[53] Pearl J. (1990). Reasoning with belief functions: An analysis of compatibility. Int. J. Approx. Reason..

[54] Wang Y., Zhang K., Deng Y. (2019). Base belief function: An efficient method of conflict management. J. Ambient. Intell. Humaniz. Comput..

[55] Smets P. (2005). Decision making in the TBM: The necessity of the pignistic transformation. Int. J. Approx. Reason..

[56] Hohle U. Entropy with respect to plausibility measures. Proceedings of the 12th IEEE International Symposium on Multiple-Valued Logic.

[57] Yager R.R. (1983). Entropy and specificity in a mathematical theory of evidence. Int. J. Gen. Syst..

[58] Dubois D., Prade H. (1985). A note on measures of specificity for fuzzy sets. Int. J. Gen. Syst..

[59] Klir G.J., Ramer A. (1991). Uncertainty in Dempster–Shafer theory: A critical re-examination. Int. J. Gen. Syst..

[60] Klir G.J., Parviz B. A note on the measure of discord. Proceedings of the Eighth International Conference on Uncertainty in Artificial Intelligence.

[61] George T., Pal N.R. (1996). Quantification of conflict in Dempster-Shafer framework: A new approach. Int. J. Gen. Syst..

[62] Jousselme A.L., Liu C., Grenier D., Bosse E. (2006). Measuring ambiguity in the evidence theory. IEEE Trans. Syst. Man, Cybern. Part A Syst. Hum..

[63] Deng Y. (2016). Deng entropy. Chaos Solitons Fractals.

[64] Jirousek R., Shenoy P.P. (2018). A new definition of entropy of belief functions in the Dempster-Shafer theory. Int. J. Approx. Reason..

[65] Pan L., Deng Y. (2018). A New Belief Entropy to Measure Uncertainty of Basic Probability Assignments Based on Belief Function and Plausibility Function. Entropy.

[66] Wu S., Tang Y. A new evidence reliability coefficient for conflict data fusion and its application in classification. Proceedings of the 2021 IEEE International Conference on Systems, Man, and Cybernetics (SMC).

[67] Zhang J., Deng Y. (2017). A method to determine basic probability assignment in the open world and its application in data fusion and classification. Appl. Intell..

[68] Lohweg V., Dörksen H. (2012). banknote authentication Data Set. Center for Machine Learning and Intelligent Systems.

[69] Asuncion A., Newman D. (2007). UCI Machine Learning Repository.

[70] Zadeh L.A., Klir G.J., Yuan B. (1996). Fuzzy Sets, Fuzzy Logic, and Fuzzy Systems: Selected Papers.

[71] Fisher R.A. (1936). The use of multiple measurements in taxonomic problems. Ann. Eugen..

[72] Halpern J.Y., Fagin R. (1992). Two views of belief: Belief as generalized probability and belief as evidence. Artif. Intell..

[73] Abellán J., Bosse E. (2018). Drawbacks of Uncertainty Measures Based on the Pignistic Transformation. IEEE Trans. Syst. Man Cybern. Syst..

[74] Abellán J. (2017). Analyzing properties of Deng entropy in the theory of evidence. Chaos Solitons Fractals.

[75] Moral-García S., Abellán J. (2020). Critique of modified Deng entropies under the evidence theory. Chaos Solitons Fractals.

